# Interpretation and Misinterpretation of Medical Abbreviations Found in Patient Medical Records: A Cross-Sectional Survey

**DOI:** 10.7759/cureus.44735

**Published:** 2023-09-05

**Authors:** Dineth C Jayatilake, Samson O Oyibo

**Affiliations:** 1 General Medicine, Peterborough City Hospital, Peterborough, GBR; 2 Diabetes and Endocrinology, Peterborough City Hospital, Peterborough, GBR

**Keywords:** interpretation, cross-sectional survey, multidisciplinary team, ambiguity, alternative definitions, patient medical records, medical errors, medical abbreviations

## Abstract

Introduction

Medical abbreviations are used in patient medical records across all departments within the hospital setting and upon discharge. Abbreviations can have more than one contradictory or ambiguous definition, which can result in errors in communication due to misunderstanding or misinterpretation. Modern patient care is multidisciplinary, so there should be no room for ambiguity in patient medical records. Therefore, the aim of this survey was to assess individual interpretations and misinterpretations of a list of medical abbreviations found in patient medical records, and thereby increase awareness of the growing use of non-standard abbreviations.

Materials and methods

In this cross-sectional survey, anonymized questionnaires containing a list of 20 abbreviations were given to a convenience sample of consultant physicians, doctors-in-training, and nurses, all of whom are involved in the day-to-day use of patient medical records. Volunteers were asked to define each abbreviation in full. A provided definition was either the intended definition (given a score of one) or completely different in terms of text and meaning (alternative definition). The intended definitions, alternative definitions, and number of abbreviations that were defined by at least 50% of volunteers were collated. Abbreviations that had more than 50% of volunteers providing the intended definition, were regarded as “generally accepted” abbreviations. Volunteers were assured that this was not a test of knowledge and that questionnaires were completely anonymized.

Results

In total, 46 volunteers completed questionnaires. Volunteers consisted of 15 nurses, 15 doctors-in-training, and 16 consultant physicians. The number of volunteers who provided the intended definition for each abbreviation ranged from zero to 87%, depending on the abbreviation. Only four out of 20 abbreviations (20%) had more than 50% of volunteers providing the intended definition and thus regarded as “generally accepted”. The maximum score achieved among the volunteers was 12 out of 20 (60%), and the minimum score achieved was 2 out of 20 (10%). The overall mean score achieved by the volunteers was 6.39 out of 20 (32%). Only one-quarter of the volunteers achieved a score above 50%. Additionally, 75% of the abbreviations had one or more (one to seven) alternative definitions.

Conclusions

This survey demonstrated that non-standard medical abbreviations used in patient medical records were being misunderstood or misinterpreted. A majority of abbreviations were not recognized among user groups. Additionally, three-quarters of abbreviations had one or more alternative definitions. Healthcare institutions should encourage the reporting of errors arising from the usage of abbreviations, and introduce initiatives to discourage the use of non-standard abbreviations in patient medical records.

## Introduction

The use of medical abbreviations has been in practice since the development of mainstream medicine. Medical abbreviations are used in all departments within the hospital setting and on discharge [1]. With healthcare professionals making up their own abbreviations, there are a growing number of non-standard abbreviations being used in patient medical records and prescriptions. People use abbreviations to save time, to fit words or phrases into small spaces, or to avoid the possibility of misspelling words. However, abbreviations are sometimes misunderstood, misread, or misinterpreted. Some abbreviations can have more than one contradictory or ambiguous definition [2]. 

Previous questionnaire-based studies have demonstrated that the use of abbreviations in patient medical records by doctors and nurses is high, and there is significant variability in the interpretation of these abbreviations among both professionals [3,4]. A study looking at the use of abbreviations in surgical note-keeping demonstrated that abbreviations are being used regularly and often inappropriately. Over 90% of surgical notes had at least one abbreviation, and at no time was the abbreviation ever fully defined [5]. 

The association between the use of abbreviations in medical prescribing and the potential for harm to the patient is well established [6]. The use of electronic prescribing has reduced prescription-induced medical errors by stopping the use of non-standard abbreviations during prescribing [7]. However, it is only recently that attempts have been made to control the use of abbreviations in patient medical records. Standard lists of “use” and “do not use” abbreviations have been created, and some healthcare institutions have created their own specialized lists of abbreviations that can be used in patient medical records [8,9]. 

While there are anecdotal examples of medical abbreviations resulting in medical errors, the potential for harm to the patient from improper communication due to medical abbreviations cannot be understated. Additionally, patient care is multidisciplinary, so there should be no room for ambiguity in patient medical records. Therefore, the aim of this survey was to assess individual interpretations and misinterpretations of a list of medical abbreviations found in patient medical records, and thereby increase awareness of the growing use of non-standard abbreviations in patient medical records. Findings from this survey could provide impetus to limit the use of non-standard abbreviations in patient medical records and thus limit the potential for harm to patients. 

## Materials and methods

Ethics approval was sought through the Research & Development Department of our institution. This cross-sectional survey did not require ethical approval. It was registered with our Quality, Governance and Compliance Department as part of a Service Evaluation Project. Verbal consent was obtained from all volunteers. Volunteers were assured of strict anonymity and confidentiality during this study.

This was a one-day cross-sectional survey. Anonymized questionnaires containing a list of 20 abbreviations were given to a convenience sample of consultant physicians, doctors-in-training, and nurses who rely on accurate medical record-keeping when contributing to patient care. Volunteers were from 10 medical wards and were chosen because they were available and willing to participate on the day. The abbreviations were preselected from a random set of patient medical records; chosen based on the fact that their definitions were unknown to any one of the study organizers. The intended definition of each abbreviation was derived from the healthcare workers who used them in the medical records. Volunteers were therefore asked to define each abbreviation in full. Volunteers were given 15 minutes to complete a questionnaire without any conferring. If they could not define the abbreviation, they could simply put “do not know”. Volunteers were assured that this was not a test of knowledge. 

Analysis

A provided definition was either the intended definition or an alternative definition, which was completely different in terms of text and meaning. For each volunteer, the number of abbreviations paired with their intended definition was collated (a score of one mark was given for each). For each abbreviation, the number of volunteers who provided the intended definition was collated. The number of alternative definitions for each abbreviation was also collated. For an abbreviation to be regarded as “generally accepted”, it was arbitrarily expected that at least 50% of volunteers would provide the intended definition for that abbreviation. 

## Results

We had 46 volunteers who each completed a questionnaire. Volunteers were made up of 15 nurses, 15 doctors-in-training, and 16 consultant physicians. All the volunteers had regular access to patient medical records while contributing to patient care. 

**Figure 1:**
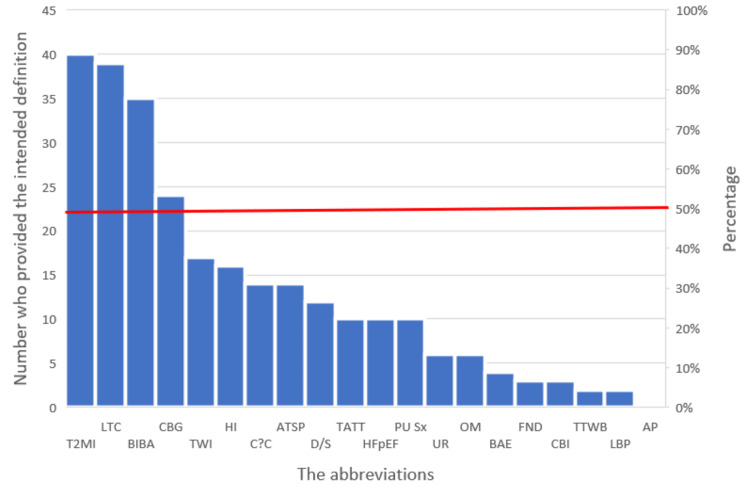
Abbreviations and number of volunteers who provided the intended definitions The left y-axis represents the number of volunteers who provided the intended definition for each abbreviation. The right y-axis represents the percentages corresponding to the numbers on the left y-axis. The x-axis represents the abbreviations in descending order of the number of volunteers who provided the intended definitions. The horizontal red line represents the point at which 50% of the volunteers provided the intended definition for a given abbreviation. Only four abbreviations had numbers above this line.

Table 1 shows the list of abbreviations with intended definitions. Figure 1 shows the number of volunteers who wrote down the intended definition for each abbreviation. The abbreviation “T2MI” had the maximum number of volunteers who wrote the intended definition (Type 2 Myocardial Infarction), and this was 40 volunteers (86.96%). The abbreviation “AP” had the minimum number of volunteers who wrote the intended definition (Abdominal Pain), with a response of zero. Only four of the 20 abbreviations (T2MI, CBG, BIBA, and LTC) had more than 50% of volunteers providing the intended definition; and therefore regarded as “generally accepted” (Figure 1).

**Table 1:** Abbreviations and intended definitions

Abbreviation	Intended definition
FND	Focal Neurology Detected
UR	Urinary Retention
T2MI	Type 2 Myocardial Infarction
D/S	Discharge Summary
C?C	Collapse Query Cause
CBG	Capillary Blood Glucose
BIBA	Brought In By Ambulance
TWI	T-Wave Inversion
TATT	Tired All The Time
LBP	Lower Back Pain
OM	Osteomyelitis
TTWB	Toe Touch Weight Bearing
AP	Abdominal Pain
HFpEF	Heart Failure with Preserved Ejection Fraction
ATSP	Asked To See Patient
CBI	Continue Bladder Irrigation
LTC	Long Term Catheter
PU S_X_	Symptoms on Passing Urine
HI	Head Injury
BAE	Bilateral Air Entry

One point was allocated for each intended abbreviation provided on the questionnaire. Table 2 shows the mean (range) scores out of 20 obtained by the three healthcare groups (consultant physicians, doctors-in-training, and nurses) for writing down the intended definitions for the list of abbreviations. The maximum score achieved among the volunteers was 12 out of 20 (60%), and the minimum score achieved was 2 out of 20 (10%). 

The overall mean score achieved by the volunteers was only 6.39 out of 20 (32%). Only 25% of the volunteers achieved a score above 10 out of 20; therefore, three-quarters of the group were unable to provide the intended definition for half of the abbreviations on the list.

**Table 2:** Mean score on the questionnaires for the three groups

Group	Number of volunteers	Mean (range) score out of 20	
		Numbers	Percentages (%)
Nurses	15	4.6 (2-9)	23.0 (10-45)
Doctors-in-training	15	8.27 (5-12)	41.35 (25-60)
Consultant physicians	16	7.5 (2-12)	37.5 (10-60)

Alternative definitions 

Any other definition written down in place of the intended definition (different in terms of text and meaning) was labelled as an alternative definition. Table 3 demonstrates the alternative definitions and the number of volunteers who wrote down these for each abbreviation. In total, 15 of the 20 abbreviations (75%) had one or more alternative definitions. The abbreviation AP had the highest number of alternative definitions (seven). Second to that was the abbreviation FND, which had five alternative definitions. 

**Table 3:** Abbreviations and their alternative definitions Alternative definitions and the number of volunteers (%) who provided them.

Abbreviation	Alternative definition	Number of volunteers who provided this definition (%)
FND	Functional Neurological Disorder	17 (36.96%)
	Full Name, Signature, Date	1 (2.17%)
	Fine Needle	1 (2.17%)
	Fundus	1 (2.17%)
	Funded Nursing Discharge	1 (2.17%)
UR	Urea	5 (10.87%)
	Upper Respiratory	1 (2.17%)
T2MI	Type 2 Diabetes Mellitus	3 (6.52%)
D/S	Deputy Sister	3 (6.52%)
	Dextrose Saline	3 (6.52%)
	Diagnosis/Symptoms	1 (2.17%)
C?C	Query Cause	1 (2.17%)
	Chronic	1 (2.17%)
	Consultant To Consultant Review	1 (2.17%)
CBG	Capillary Blood Gas	14 (30.43%)
	Coronary Artery Bypass Graft	1 (2.17%)
BIBA	-	-
TWI	Twilight	1 (2.17%)
	Time Waiting In	1 (2.17%)
TATT	-	-
LBP	Lying Blood Pressure	26 (56.52%)
	Low Blood Pressure	8 (17.39%)
OM	Once in the Morning	22 (47.83%)
	Old Man	1 (2.17%)
	Omit	1 (2.17%)
	Otitis Media	1 (2.17%)
	Over Medicated	1 (2.17%)
TTWB	Total Weight Bearing	2 (4.35%)
AP	Anterior Posterior View	16 (34.78%)
	Abdominopelvic	3 (6.52%)
	Anterior Projection	2 (4.35%)
	Acute Physician	2 (4.35%)
	Adynamic Precordium	1 (2.17%)
	Allied Practitioner	1 (2.17%)
	Advance Practice	1 (2.17%)
HFpEF	-	-
ATSP	-	-
CBI	Continuous Bladder Infection	1 (2.17%)
	Capillary Insulin	1 (2.17%)
	Cerebral Brain Injury	1 (2.17%)
LTC	Lower Urinary Tract Catheter	1 (2.17%)
PU S_X_	Pressure Ulcer Symptoms/Surgery	4 (8.70%)
	Passed Urine	3 (6.52%)
	Pelvic Ureter Surgery	2 (4.35%)
	Pressure Ulcer Sore Sacrum	1 (2.17%)
	Pus in Urine Specimen	1 (2.17%)
HI	-	-
BAE	Bronchial Artery Embolization	1 (2.17%)
	Bronchial Asthma Exacerbation	1 (2.17%)

The abbreviation “LBP” had the highest number of volunteers (56.52%) who provided an alternative definition (Lying Blood Pressure) instead of the intended definition (Low Back Pain). Second was the abbreviation “OM”, which had 47.83% of volunteers who provided an alternative definition (One in the Morning) instead of the intended definition (Osteomyelitis). In third and fourth place were the use of the alternative definitions “Functional Neurological Disorder” and “Capillary Blood Gas” for the abbreviations “FND” and “CBG” instead of the intended definitions (Focal Neurology Detected and Capillary Blood Glucose), respectively.

## Discussion

We administered questionnaires containing a list of 20 abbreviations used in patient medical records to a group of doctors and nurses involved in patient care in a secondary care hospital. Volunteers were asked to provide the definitions of the abbreviations presented. This survey demonstrated that none of the volunteers were able to provide the intended definitions for all 20 abbreviations on the list, and none of the abbreviations had the intended definition provided by every volunteer. Less than 25% of volunteers knew more than 50% of the abbreviations presented to them. Only 20% of the abbreviations had more than 50% of volunteers able to provide the appropriate intended definition: therefore, only these abbreviations (T2MI, CBG, BIBA, and LTC) could be regarded as “generally accepted”. Additionally, 75% of the abbreviations had one or more alternative definitions and were therefore ambiguous. 

This survey revealed several examples of how multiple interpretations of a single abbreviation can lead to miscommunication and medical errors, for example, the abbreviation "T2MI" for Type 2 Myocardial Infarction being interpreted as Type 2 Diabetes Mellitus by some volunteers; the abbreviation "CBG" for Capillary Blood Glucose being interpreted as Capillary Blood Gas by a significant number of volunteers; the abbreviation "FND" for Focal Neurology Detected being interpreted as Functional Neurological Disorder by several volunteers; and the abbreviation "TTWB" for Toe Touch Weight Bearing being interpreted as Total Weight Bearing by some volunteers.

The results of this survey mirror that of previously published studies. In a study evaluating the understanding of common medical abbreviations among doctors from several departments in an academic hospital, the findings suggested that the understanding of medical abbreviations across medical departments is below standard. This was even worse for non-standard abbreviations [10]. The authors went on to suggest that the use of non-standard medical abbreviations should be discouraged [10]. Another study of common abbreviations used in surgical inpatient admissions demonstrated similar poor results and concluded that the use of an unambiguous and approved list of abbreviations is required to ensure good communication in patient care [11]. A recent Danish study found that many abbreviations had multiple meanings, and that writing a sentence with abbreviations saved 20 seconds, while comprehension of an abbreviated sentence took an extra 12-85 seconds [12]. The authors went on to suggest solutions such as: embracing and expanding the use of abbreviations, the introduction of artificial intelligence to interpret abbreviations, or the use of speech recognition software in all Danish hospitals [12]. A large Australian study, using an abbreviation extracting software revealed that one-third of abbreviations used in general medical discharge summaries were ambiguous [13]. Although studies evaluating the use of non-standard abbreviations are few, they all portray the same message. More than 50% of medical abbreviations, especially non-standard ones, are ambiguous, and should not be used in patient medical records. 

During this survey we received several anecdotal reports of some abbreviations being misunderstood or misinterpreted, resulting in minor medical errors; however, such events were never officially reported. We believe that abbreviations used in patient medical records should be standardized and fully accepted by everyone who has access to the records for the provision of patient care. Patient care is multidisciplinary, thus multiple healthcare groups have access to patient medical records in hospitals. Healthcare staff should not have to keep looking up the meaning or asking another professional for the full meaning of abbreviations used in patient medical records; this is time-consuming. Medical errors and near misses arising from the use of abbreviations should be officially reported. 

Survey limitations 

Firstly, the abbreviations were assessed out of context by volunteers. Volunteers may have been able to define the abbreviations if presented within context. Further studies should be designed to assess the understanding of abbreviations presented within context. Secondly, the intended definition of an abbreviation was derived beforehand from the healthcare worker who used it in the patient's medical records. Therefore, it is possible that the derived definition was an alternative definition from the start. This will be a continuous problem with the growing number of non-standard abbreviations. Thirdly, the volunteers were made up of doctors and nurses who may have been able to recognize abbreviations specific to their working group. We only included volunteers working within the Department of Medicine. Fourthly, as this was a convenience sample, a sampling bias cannot be completely ruled out. The sample may not be typical of the total doctors and nurses workforce in our institution, and findings may not apply to other healthcare institutions. Patient medical records are multidisciplinary and accessed by multiple healthcare groups and across multiple departments. Therefore, further studies using random sampling methods should include a more diverse group of healthcare professionals from different settings and departments. 

## Conclusions

There is a growing use of non-standard abbreviations in medical practice. A majority of abbreviations are not recognized among user groups and a majority have multiple alternative definitions. The use of abbreviations in healthcare can lead to poor communication and misinterpretation, which can result in medical errors. When writing an abbreviation, one should consider the following points: (i) Is this a standard abbreviation? (ii) Will everyone, including the patient be able to interpret the abbreviation? (iii) Is abbreviating necessary? (iv) Will abbreviating really save time? 

Medical errors or near misses resulting from the use of abbreviations in patient medical records should be officially reported. This will help in understanding the extent of the problem and in formulating solutions. Additionally, healthcare institutions should provide clear policies on the use of abbreviations, and introduce initiatives to discourage the use of non-standard abbreviations, such as abbreviation-free periods or standard abbreviations awareness programs.

## References

[1] Tariq RA, Sharma S (2023). Inappropriate medical abbreviations. StatPearls.

[2] Pennsylvania Patient Safety Reporting System (2005). Abbreviations: a shortcut to medication errors. PA-PSRS Patient Safety Advisory.

[3] Soto-Arnáez F, Sebastián-Viana T, Carrasco-Garrido P, Fernández-de-Las-Peñas C, Parás-Bravo P, Palacios-Ceña D (2019). A descriptive study of the knowledge of nurses and doctors of clinical abbreviations in hospital discharge reports. Enferm Clin (Engl Ed).

[4] Koh KC, Lau KM, Yusof SA, Mohamad AI, Shahabuddin FS, Ahmat NH, Teh PC (2015). A study on the use of abbreviations among doctors and nurses in the medical department of a tertiary hospital in Malaysia. Med J Malaysia.

[5] Collard B, Royal A (2015). The use of abbreviations in surgical note keeping. Ann Med Surg (Lond).

[6] Dooley MJ, Wiseman M, Gu G (2012). Prevalence of error-prone abbreviations used in medication prescribing for hospitalised patients: multi-hospital evaluation. Intern Med J.

[7] Cheung S, Hoi S, Fernandes O, Huh J, Kynicos S, Murphy L, Lowe D (2018). Audit on the use of dangerous abbreviations, symbols, and dose designations in paper compared to electronic medication orders: A multicenter study. Ann Pharmacother.

[8] Grossman Liu L, Grossman RH, Mitchell EG, Weng C, Natarajan K, Hripcsak G, Vawdrey DK (2021). A deep database of medical abbreviations and acronyms for natural language processing. Sci Data.

[9] (2023). Institute for Safe Medication Practices (ISMP). ISMP list of error-prone abbreviations, symbols, and dose designations. https://www.ismp.org/recommendations/error-prone-abbreviations-list.

[10] Sherriff A, Ally H, Mahomed W, Rae H, Schanknecht R, Sealanyane S, Joubert G (2017). The understanding of medical abbreviations across different medical departments in a South African hospital setting. Commun Med.

[11] Sinha S, McDermott F, Srinivas G, Houghton PW (2011). Use of abbreviations by healthcare professionals: what is the way forward?. Postgrad Med J.

[12] Zetner DB, Zetner DB, Glerup SB (2022). Christmas article: Brevity is not to the point - a study of abbreviations. Ugeskr Laeger.

[13] Holper S, Barmanray R, Colman B, Yates CJ, Liew D, Smallwood D (2020). Ambiguous medical abbreviation study: challenges and opportunities. Intern Med J.

